# DOVIS: an implementation for high-throughput virtual screening using AutoDock

**DOI:** 10.1186/1471-2105-9-126

**Published:** 2008-02-27

**Authors:** Shuxing Zhang, Kamal Kumar, Xiaohui Jiang, Anders Wallqvist, Jaques Reifman

**Affiliations:** 1 Biotechnology HPC Software Applications Institute, Telemedicine and Advanced Technology Research Center, US Army Medical Research and Materiel Command, Fort Detrick, MD 21702, USA; 2 Department of Experimental Therapeutics, MD Anderson Cancer Center, 1515 Holcombe Blvd, Unit 36, Houston, TX 77030, USA

## Abstract

**Background:**

Molecular-docking-based virtual screening is an important tool in drug discovery that is used to significantly reduce the number of possible chemical compounds to be investigated. In addition to the selection of a sound docking strategy with appropriate scoring functions, another technical challenge is to *in silico *screen millions of compounds in a reasonable time. To meet this challenge, it is necessary to use high performance computing (HPC) platforms and techniques. However, the development of an integrated HPC system that makes efficient use of its elements is not trivial.

**Results:**

We have developed an application termed DOVIS that uses AutoDock (version 3) as the docking engine and runs in parallel on a Linux cluster. DOVIS can efficiently dock large numbers (millions) of small molecules (ligands) to a receptor, screening 500 to 1,000 compounds per processor per day. Furthermore, in DOVIS, the docking session is fully integrated and automated in that the inputs are specified via a graphical user interface, the calculations are fully integrated with a Linux cluster queuing system for parallel processing, and the results can be visualized and queried.

**Conclusion:**

DOVIS removes most of the complexities and organizational problems associated with large-scale high-throughput virtual screening, and provides a convenient and efficient solution for AutoDock users to use this software in a Linux cluster platform.

## Background

In the last several years, virtual screening has become an accepted tool in drug discovery. It has been successfully applied in a number of therapeutic programs, in particular, at the lead discovery stage, where high-throughput molecular docking can play an important role [1]. In concert with the continued need for improvements of *in silico *docking accuracies, the explosive growth of commercial and publicly available chemical databases requires computational techniques to efficiently implement docking protocols and rapidly screen millions of compounds in a timely fashion. Here, we are focusing on the techniques to enable large scale docking using Linux-based HPC platforms.

Several commercial docking programs, such as Glide [2], LigandFit [3] and FlexX [4], can distribute docking jobs to computers over the network. However, protocols that can seamlessly dock millions of compounds and capture the top percentage of high-scoring ligands are not standard. There are two major requirements for such a protocol running on a Linux cluster: (1) the ability to launch parallel docking jobs through a queuing system; and (2) the ability to process millions of compounds in a reasonable time. Since the latter requirement may call for hundreds of central processing units (CPUs) working simultaneously, the protocol should effectively handle the associated data flow through the file system without affecting the performance of the cluster. In this note, we describe a Linux cluster-based protocol using AutoDock [5] as the docking engine.

AutoDock is a widely used docking program developed at the Scripps Research Institute. Application of AutoDock requires several separate pre-docking steps, e.g., ligand preparation, receptor preparation, and grid map calculations, before the actual docking process can take place. Existing tools, such as AutoDock Tools (ADT) [6] and BDT [7], integrate individual AutoDock steps within a graphical user interface (GUI), and provide automated features for docking runs. However, they do not contain the capability to effectively process millions of compounds in a single execution. At the AutoDock Website [8], there is a tutorial with scripts to teach users how to use AutoDock and how to employ UNIX commands to perform virtual screening. In the tutorial, first a user needs to manually prepare the receptor, ligands, energy grids and a list of ligands names; then every compound in the list is looped through by a shell script, where the compound can be docked either by executing AutoDock commands inside the loop or by submitting the corresponding commands to a queuing system. This approach works well for thousands of compounds, however, it may not work for millions of compounds due to the limitations of the file and queuing systems. Concurrently, AutoDock has also been used in grid-computing projects, FightAIDS@Home [9] and WISDOM [10]. In a grid-computing infrastructure, tens to hundreds of thousand CPUs are used to setup a computational grid. Through specialized middleware, servers in a grid can schedule jobs, send applications and data to a CPU, and retrieve results. Because of the large number of CPUs involved, the computational power of a grid is phenomenal. For example, in the WISDOM project, 10 million docking experiments were performed using AutoDock in 20 days. However, due to the nature of its infrastructure, the job success rate was 65% and the percentage of time spent running the application was ~50%. On the other hand, using a dedicated HPC platform (e.g., a 128-node Linux cluster) with specialized software tools for AutoDock, it is possible to screen millions of compounds in a reasonable time at a much higher job success rate and CPU efficiency.

Here, we describe a DOcking-based VIrtual Screening (DOVIS) pipeline, based on AutoDock (version 3), where Perl and shell scripts are used to integrate executables and scripts from ADT, OpenBabel [11] and AutoDock. This implementation has the following advantages: (1) a scalable parallelization scheme for AutoDock integrated with queuing systems, such as the Load Sharing Facility (LSF), Platform Computing Inc. (Ontario, Canada) and the Portable Batch System (PBS), Altair Grid Technologies (Troy, MI); (2) a protocol to retain user-specified top percentage of docked ligands based on their docking scores; (3) a X-window's-based GUI for users to specify docking parameters, submit docking jobs and query/visualize docked ligands; and (4) a collection of pre-processed compounds from the ZINC database [12] and the National Cancer Institute (NCI) diversity dataset [13] in native AutoDock pdbq format.

## Implementation

A high-throughput screening campaign typically has many more ligands (millions) than the number of CPUs (hundreds) available. In this case, a straightforward approach to process the ligands is via a parallelization scheme where the input ligands (or database) are divided into *N *equal partitions corresponding to the number of CPUs available for docking. For each CPU, a single partition of ligands is docked to the target receptor. After all docking jobs are completed, the results from the *N *CPUs are consolidated and the final (user specified) top *M *scoring ligands are returned to the user for further analysis.

The input parameters for screening are specified via a GUI, which creates one master parameter file that is used to drive all related scripts and programs. Inside DOVIS there are three distinct implementation steps, pre-docking, parallel docking, and post-docking, which are integrated with a queuing system.

### Pre-docking

In this step, the receptor is converted to the native AutoDock format and the ligands are partitioned into *N *files. DOVIS accepts receptors with all hydrogen atoms specified in standard pdb or mol2 format. A Python script, "prepare_receptor.py" from ADT, is used to convert the receptor into the pdbqs format required by AutoDock. The acceptable input formats for ligands include sd, mol2, and pdbq. When the ligand is provided in the sd format, it is converted into mol2 by OpenBabel. The mol2 files are then partitioned into *N *files with roughly the same number of molecules in each file. If the input ligands are provided as pre-processed pdbq files, the list of ligand file names is simply partitioned into *N *lists of roughly equal length.

### Parallel Docking

From the previous step, either a ligand file (containing multiple ligands) or a list of ligands is assigned to each CPU, where the ligands in each set are separately processed and docked to the receptor, one ligand at a time per CPU. For ligands in the mol2 format, a Python script, "prepare_ligand.py" from ADT, is used to generate the corresponding pdbq file. We pre-compute energy grid maps for all possible ligand atom types at the beginning of each parallel job. Thus, any grid map needed by AutoDock can be directly loaded without a separate calculation. After each docking, the docking log-file (dlg) is parsed by a Perl script to extract the "estimated free energy of binding," which is used as the criterion to select the top *M *scoring dlg files from each CPU.

During testing, we found that there are more than 10 file operations and over 10 Mb of data flow associated with the docking of each ligand. If a Network File System (NFS) is used with more than 100 CPUs running concurrently, the data flow degrades the performance of the entire Linux cluster. To overcome this problem, DOVIS provides the option to use the local disk drive on each node or CPU to store the energy grid maps and other intermediate files.

### Post-docking

After all docking jobs are completed, a Perl script is used to combine all saved results from each CPU and to collect the top *M *scoring ligands from the consolidated list as the final result. The dlg files of all selected top-scoring ligands are collected and compressed into a directory.

### Queue Integration

On large Linux clusters with distributed memory, it is essential to integrate DOVIS with a queuing system. With the job dependency support of a queuing system, the three steps discussed above can be sequentially executed and parallel jobs can be automatically launched. For the parallel docking job, at each CPU, the same executables are used to process different ligands. This computational strategy is most efficiently handled by queuing systems that support the functionality to bundle many single-processor jobs using the same executables into a single job, typically referred to as Job Array. Thus, DOVIS is suitable for any queuing system that supports Job Array and job dependency. Currently, we have integrated DOVIS with the LSF and the PBS queuing systems.

In order to run DOVIS on Linux clusters without a queuing system, we provide a version of DOVIS that uses multi-threading to run parallel docking jobs. This scheme is especially suitable for shared-memory Linux clusters.

There is another issue related to shared queuing systems. Usually, every job in the queue has a runtime limit. Once the limit is reached, the job is terminated. When docking large number of ligands, Job Array may not be able to complete all tasks within the allowed runtime limit. Therefore, we implemented a restart function, which tracks the progress of each CPU and gives the user the option to manually restart DOVIS from where it left off.

### Graphic User Interface

A GUI was developed for DOVIS using Java Swing to provide a convenient way to specify the target receptor, the ligand database and the docking parameters. Using the GUI, users are also able to submit the job to the queuing system. In addition, the JMol [14] molecular viewer is embedded in the GUI to enable visualization of docked ligands with the target receptor.

## Results

In order to build a ligand database for testing and scientific research purpose, we pre-processed the ZINC database (version 5, 2.07 million compounds) and the NCI diversity dataset (1,990 compounds) into AutoDock pdbq format. Using the pre-processed ZINC database, we tested how many ligands DOVIS could dock to a receptor per day with varying numbers of CPUs. We performed tests with up to 128 CPUs on a Linux cluster and observed a near-linear speedup as a function of number of CPUs, for the ZINC database. This indicates that our implementation achieves near-optimal performance. In addition, we carried out the virtual screening of the ZINC database against the ricin A chain (267 amino acids) as a receptor target with 256 CPUs. The task was completed in 12 days, corresponding to ~700 ligands per CPU per day, with two manual restarts due to a four-day runtime limit in our queuing system. See additional file 1 for the detailed parameter choices.

## Discussion

We developed DOVIS as a utility software to automate docking jobs with AutoDock. It can reliably screen millions of compounds against a receptor and automatically save the top percentage of high-scoring hits. The parallelization scheme employed here provides a straightforward approach to such a problem. When all CPUs are started around the same time, this scheme works well and all CPUs shall complete their jobs at about the same time. However, when the queuing system does not allow all CPUs to be launched at the same time, with the equal number of ligands assigned to each CPU, the efficiency of DOVIS is compromised. This may become a problem with a queuing system when a large number of CPUs are requested for a calculation. To solve this problem, the number of ligands assigned to each CPU should be re-distributed in a way that the CPUs launched earlier shall docked more ligands than the CPUs launched later. Currently, we are working on an improvement to address this issue.

## Conclusion

Using AutoDock as its docking engine, DOVIS provides an automated parallel docking package that is integrated with a queuing system. This application is suitable for conducting large-scale high-throughput virtual screening on Linux cluster platforms. The DOVIS package is freely available (see additional file 1 and 2 for detailed information).

## Authors' contributions

SZ implemented and tested the software. KK implemented the GUI and tested the software. XJ participated in the software design and testing, and drafted the manuscript. JR and AW conceived the project, participated in the software design and project coordination. All authors read and approved the final manuscript.

## Supplementary Material

Additional file 1 Supplementary_material_DOVIS_paper. This file describes the software packages required to setup DOVIS, the AutoDock parameters used to run a virtual screening reported in the paper, and the DOVIS package installation options. Click here for file

Additional file 2 DOVIS_alpha_Release.tar.gz. This file contains the DOVIS package (DOVIS_alpha), release note and user manual. Click here for file
