# Supplementary material for: DOVIS: an implementation for high-throughput virtual screening using AutoDock
Source: BMC Bioinformatics. 2008 Feb 27;9:126. doi: 10.1186/1471-2105-9-126 (PMC2267697; doi:10.1186/1471-2105-9-126)
Supplement: Additional file 1 — Supplementary_material_DOVIS_paper. This file describes the software packages required to setup DOVIS, the AutoDock parameters used to run a virtual screening reported in the paper, and the DOVIS package installation options. [file 1471-2105-9-126-S1.doc]

Supplementary material for

DOVIS: an Implementation for High-throughput Virtual Screening using AutoDock

1. Software packages
   1. AutoDock

The software AutoDock was obtained from The Scripps Research Institute (<http://autodock.scripps.edu/>). The package includes AutoDock, AutoGrid, AutoTors and related scripts. We modified a header file (autocomm.h) in both AutoDock and AutoGrid to increase the number of atom types and atoms maps allowed. AutoGrid and AutoDock were re-compiled to build the modified executables. The values that we changed in our version of autocomm.h are:

MAX_MAP 18

ATOM_MAP 16

NATOMTYPES 10

The atoms that we computed in our gpf file are “CANOSPHXFMZLcbIf”. The modified autocomm.h and our gpf file are available at <http://www.bioanalysis.org/downloads/DOVIS_alpha.tar.gz> .

- 1. ADT

The software ADT was obtained from The Scripps Research Institute (<http://autodock.scripps.edu/resources/adt>).

- 1. OpenBabel

OpenBabel is an open source, GNU GPL licensed software ([http://openbabel.sourceforge.net](http://openbabel.sourceforge.net/)).

- 1. LSF

Load Share Facility (LSF) is a queuing system from Platform Computing Inc ([http://www.platform.com](http://www.platform.com/Products/Platform.LSF.Family)).

- 1. PBS

Portable Batch System (PBS) is a queuing system from Altair Grid Technologies ([http://www.openpbs.org](http://www.openpbs.org/)).

- 1. JMOL

The software JMOL is an open source molecular viewer (<http://jmol.sourceforge.net/>).

- 1. DOVIS pipeline

The scripts, parameter files and Java based GUI for the DOVIS pipeline are available at <http://www.bioanalysis.org/downloads/DOVIS_alpha_Release.tar.gz> .

- 1. ZINC database

The ZINC database is available at [http://zinc.docking.org/](http://zinc.docking.org/ ). Due to the large file size of the processed ZINC database (version 5) in pdbq format, we can not be it online for download. However, we will distribute it on DVD per request.

1. AutoDock virtual screening parameters

The virtual screening of the ZINC database against the ricin A chain was performed using the default parameters in AutoGrid and AutoDock except the following:

npts 60 60 60 #num.grid points in xyz

ga_pop_size 200 # number of individuals in population

ga_num_evals 250000 # maximum number of energy evaluations

ga_run 10 # do this many GA or LGA runs

1. DOVIS package options

In the current release, DOVIS runs on Linux computers. Depending on the computer setup, it can either use a queuing system (LSF or PBS), or use multi-threading for parallel dockings. The current package options are:

- 1. A Linux workstation or cluster with a queuing system (LSF or PBS queue) in place. Users should use the corresponding DOVIS_alpha_LSF or DOVIS_alpha_PBS package.
  2. A Linux workstation with multi-core (multiple CPUs in one Linux machine). Users should use the DOVIS_alpha_MT package.

The release note contains the detailed instruction on how to install the package.
